# Changes over time in HIV testing and counselling uptake and associated factors among youth in Zambia: a cross-sectional analysis of demographic and health surveys from 2007 to 2018

**DOI:** 10.1186/s12889-021-10472-x

**Published:** 2021-03-06

**Authors:** Aimé Bitakuya Heri, Francesca L. Cavallaro, Nurilign Ahmed, Maurice Mubuyaeta Musheke, Mitsuaki Matsui

**Affiliations:** 1 Department of Global Health, Nagasaki University School of Tropical Medicine and Global Health, Sakamoto 1-12-4, Nagasaki, 852-8523 Japan; 2 Institute of Child Health, University College London, 30 Guilford St, Holborn, London, WC1N 1EH UK; 3 Faculty of Public Health and Policy, London School of Hygiene and Tropical Medicine, Keppel Street, London, WC1E 7HT UK; 4 Centre for Infectious Disease Research in Zambia, Plot # 34620, Off Alick Nkhata Road, Lusaka, Zambia

**Keywords:** HIV/AIDS, HTC, HIV testing, 90–90-90, Youth, Adolescents, Sub-Saharan Africa, Zambia, Demographic health survey

## Abstract

**Introduction:**

Zambia is among the countries with the highest HIV burden and where youth remain disproportionally affected. Access to HIV testing and counselling (HTC) is a crucial step to ensure the reduction of HIV transmission. This study examines the changes that occurred between 2007 and 2018 in access to HTC, inequities in testing uptake, and determinants of HTC uptake among youth.

**Methods:**

We carried out repeated cross-sectional analyses using three Zambian Demographic and Health Surveys (2007, 2013–14, and 2018). We calculated the percentage of women and men ages 15–24 years old who were tested for HIV in the last 12 months. We analysed inequity in HTC coverage using indicators of absolute inequality. We performed bivariate and multivariate logistic regression analyses to identify predictors of HTC uptake in the last 12 months.

**Results:**

HIV testing uptake increased between 2007 and 2018, from 45 to 92% among pregnant women, 10 to 58% among non-pregnant women, and from 10 to 49% among men. By 2018 roughly 60% of youth tested in the past 12 months used a government health centre. Mobile clinics were the second most common source reaching up to 32% among adolescent boys by 2018. Multivariate analysis conducted among men and non-pregnant women showed higher odds of testing among 20–24 year-olds than adolescents (aOR = 1.55 [95%CI:1.30–1.84], among men; and aOR = 1.74 [1.40–2.15] among women). Among men, being circumcised (aOR = 1.57 [1.32–1.88]) and in a union (aOR = 2.44 [1.83–3.25]) were associated with increased odds of testing. For women greater odds of testing were associated with higher levels of education (aOR = 6.97 [2.82–17.19]). Education-based inequity was considerably widened among women than men by 2018.

**Conclusion:**

HTC uptake among Zambian youth improved considerably by 2018 and reached 65 and 49% tested in the last 12 months for women and men, respectively. However, achieving the goal of 95% envisioned by 2020 will require sustaining the success gained through government health centres, and scaling up the community-led approaches that have proven acceptable and effective in reaching young men and adolescent girls who are less easy to reach through the government facilities.

**Supplementary Information:**

The online version contains supplementary material available at 10.1186/s12889-021-10472-x.

## Background

HIV/AIDS remains a leading cause of death in low- and middle-income countries (LMICs), with around 663,000 deaths estimated in 2019 [1, 2]. In the sub-Sahara African region, home to more than two-thirds (25.7 million) of people living with HIV globally, HIV/AIDS is the fourth leading cause of death [2]. The remarkable scale-up of access to HIV prevention and care services made in the past two decades, through multiple global health initiatives such as the Global Fund to Fight Tuberculosis, HIV/AIDS, and Malaria (GFATM), and the United States President’s Emergency Plan for AIDS Relief (PEPFAR) [3], has led to a significant reduction in morbidity and mortality due to HIV/AIDS in affected populations [2]. Despite the overall progress, only minor declines in new HIV infections have been observed among young people in high-burden countries, especially for adolescent girls and young women [4, 5]. Youth (15–24 years) still account for over 30% of new HIV infections each year globally [6].

Zambia faces a generalised HIV epidemic with an estimated prevalence of 11% among adults (15–49 years), which ranks it among the ten most affected countries globally [7, 8]. Among 48,000 estimated new infections in 2018, 39% were youth; of which more than two-thirds were women [9]. National data show that around 90% of these HIV infections are the result of unprotected heterosexual intercourse [10]. Individual and contextual drivers are thought to be among the main contributors of transmission including biological, behavioural, cultural, socio-economic, and legal factors [10–14]. HIV prevention programs that comprehensively address these drivers are required to achieve substantial change in the incidence of infection, with a cascade HIV care approach being key to the reduction of HIV transmission [15, 16].

In 2014, UNAIDS launched a fast-track global strategy to end the HIV epidemic by 2030, central to which is the 90–90-90 cascade goals; namely, aiming to ensure that 90% of people living with HIV know their status, 90% of those in HIV care are initiated on antiretroviral therapy (ART), and 90% of those on ART achieve viral load suppression by 2020 [17]. HIV testing and counselling (HTC) services are therefore critical for the reduction of new infections because they constitute the entry point of this cascade of HIV care and the means through which the first step of the 90–90-90 UNAIDS goal can be achieved [18–20]. Unfortunately, the use of HTC services has generally been reported to be low among youth in Zambia, and several barriers to accessing these services have been highlighted [21–24]. In alignment with the UNAIDS fast-track strategy, the Zambian government launched the HTC Implementation Plan (2014–16), which aimed to achieve by 2015 50% HTC coverage among adults for testing and receipt of the results in the last 12 months, to guarantee that the country remains on track with the 2020 targets [25]. The ongoing current National AIDS Strategic Framework (NASF) 2017–2021 remained aligned with the UNAIDS 90–90-90 global strategy and integrated the priority given to adolescents and young people [10]; such that the related HTC guidelines recommended for adolescents (10–19 years) and adults who are sexually active or with unknown HIV status to undertake a serological HIV test at first contact with health services, 3 months later if they were negative for the first test, and repeat the test every 6 months [26, 27].

To our knowledge, no study has investigated changes in HTC uptake among youth in Zambia after the launch of the 90–90-90 fast track targets and youth-specific global initiatives (“ALL IN Initiative” and “THREE fast-track”), which were launched to boost the HIV response toward this population [28, 29]. Moreover, the uncertainty around achieving the 2020 goals for HIV/AIDS-related morbidity and mortality among youth reflects the challenges in accessing HIV prevention and treatment services and highlights the importance of understanding who is, and who is not accessing the first step of the cascade of HIV care - HTC - and associated factors, to help refine control strategies and maximise the impact of future interventions [6].

The objectives of this study were to examine changes in uptake of HTC among youth between 2007 and 2018, to explore inequity in testing uptake over time, and to identify the determinants of HTC among young women and men ages 15–24 years-old in Zambia.

## Methods

### Data source and study population

This study used three datasets from the 2007, 2013–14, and 2018 Zambian Demographic Health Surveys (DHS). The Zambia Statistics Agency conducts these nationally representative, population-based surveys using a stratified two-stage cluster sampling method [7, 24]. In this study, we included all women and men respondents ages 15–24 years old, regardless of reported sexual activity.

### Outcome variable

The primary outcome of interest was respondents reporting that they had been tested for HIV in the last 12 months and received the results.

### Determinants of HIV testing

**Fig. 1:**
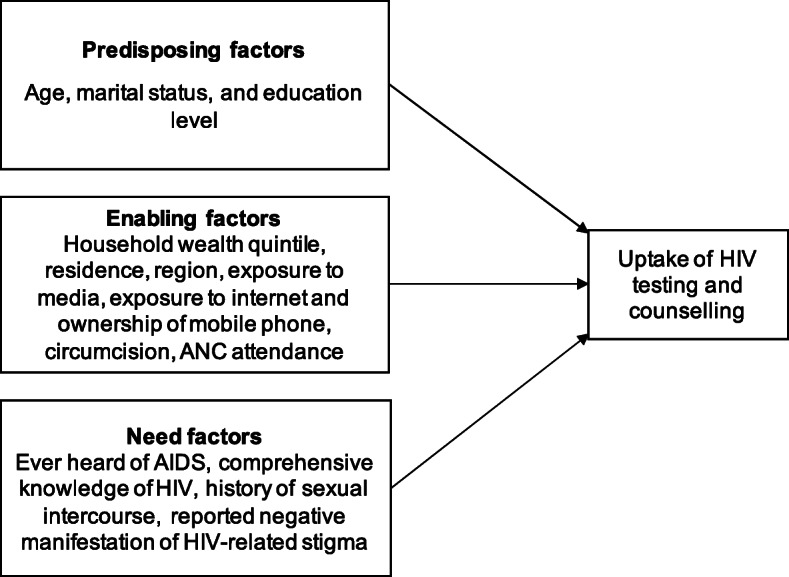
Adapted Andersen Behavioural Model of utilization of HIV testing and counselling services

Potential determinants of HIV testing and counselling were selected based on Andersen’s behavioural model which suggests three factors influencing utilisation of a health service [30, 31]; specifically, (i) predisposing factors in any condition that can enhance the risk of engaging in unprotected sexual behavior, such as age and education; (ii) enabling factors that would increase chances of greater access to HTC, such as wealth and urban residence; and (iii) required factors which would affect a perceived need for HTC, such as knowledge of HIV or being sexually active. On this basis, we identified available determinants in the DHS, shown in Fig. 1 (a full list and definitions are shown in Supplementary Table S1) [21, 32–36].

### Data analysis

For each survey we calculated the percentage of respondents tested for HIV in the last 12 months who received results; for adolescents (15–19), and young (20–24) women and men separately. To examine the extent of an antenatal care (ANC) session as a contributing factor in higher testing rates among women, we separated them into non-pregnant and pregnant groups. The testing uptake for pregnant women considered those who had a birth in the last 12 months and received their test as part of ANC. The source of HTC (in the last 12 months) was also described for each survey and each age group of women and men, separately. The numbers of annual tests conducted through each testing source were estimated by multiplying the percentage of tests reported per source multiplied by the estimated population of young women and men obtained from the World Bank population projections [37]; separately for adolescents and young adults of each gender.

We examined changes in inequity in HIV testing coverage from 2007 to 2018 separately among men and non-pregnant women. We used the Equiplot graph suggested by the International Center for Equity in Health (ICEH) to illustrate changes across time points for inequalities related to age, residence, education level, and household wealth [32, 38]. The mean coverage difference from the best-performing region was also calculated to show regional disparities in testing coverage across time points [39]. We restricted the analysis of inequity and predictors of HTC uptake among women to those non-pregnant, to avoid bias in the estimate of HIV testing coverage due to high rates observed through ANC.

Potential determinants (listed in Fig. 1) of HTC uptake were examined using the most recent survey dataset (2018). We first described respondent characteristics, then conducted bivariate analyses to identify those associated with the primary outcome; specifically, HIV testing in the last 12 months with receipt of results. After checking for collinearity and excluding variables with missing values for the outcome, all variables associated with the primary outcome (with *p* < 0.05) were considered for inclusion in the multivariate logistic regression models. The main models were run separately for men and non-pregnant women. Considering that 90% of HIV infections are reported to be transmitted through heterosexual intercourse in Zambia [10], a sub-group analysis restricted to men and non-pregnant women reported being sexually active was also conducted to identify specific determinants associated with HTC uptake in this population.

STATA SE version 16.1 (Stata Corporation, Ltd. Texas, USA) was used for analysis, and all analyses considered clustering, weighting, and stratification using the *svyset* command.

## Results

### Changes in HIV testing uptake

**Fig. 2:**
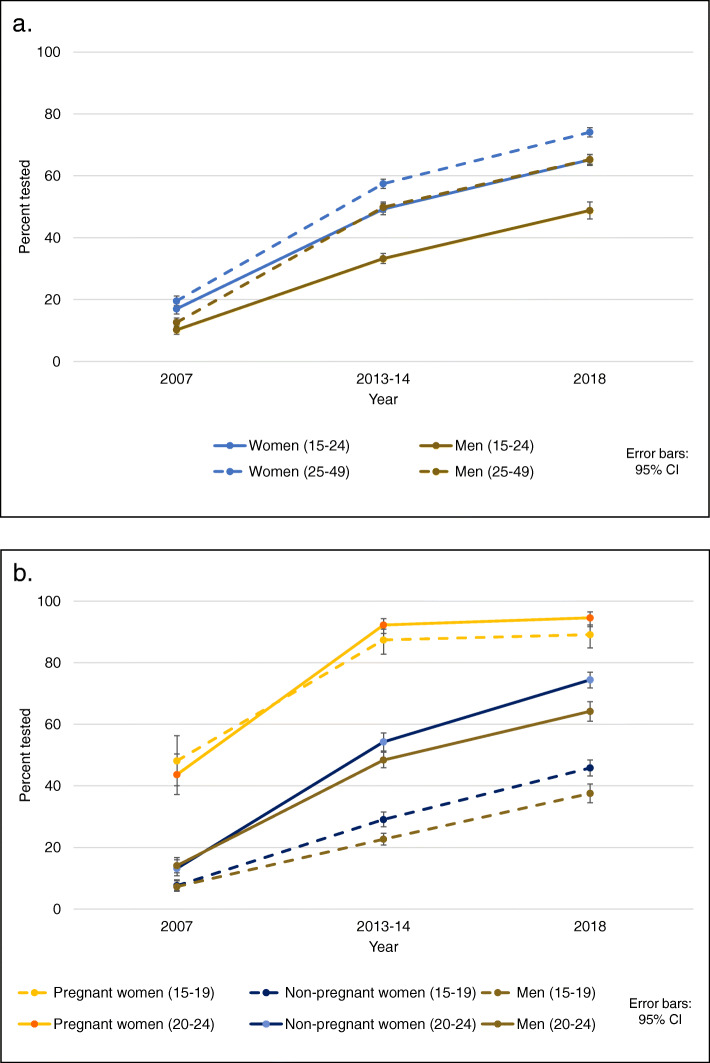
Changes in HIV testing uptake in Zambia, from 2007 to 2018: **a**. Among adults and youth tested in the last 12 months and receiving the result, **b**. among youth tested in the last 12 months and receiving the result

Overall, an improvement in HIV testing was observed between 2007 and 2018, with the percentage tested and receiving the results in the last 12 months increasing from 17 to 65% among young women, and from 10 to 49% among young men (Fig. 2a). These increases, observed in both genders and age groups (ages 15–19 and 20–24), were more pronounced between the period 2007–2013 than 2013–2018, and the absolute increase was higher among women than men (Fig. 2b). The testing rate among pregnant women, tested as part of ANC, increased considerably from 2007 to 2013 and nearly reached universal coverage for both age groups, with almost no change between 2013 and 2018. Figure 2b also shows that HIV testing uptake among non-pregnant women was much lower in all age groups than among women who received a test as part of ANC. Moreover, women not recently pregnant had almost the same testing coverage as men between 2007 and 2013, for both age groups. Nevertheless, a small difference between genders was noted from 2013 to 2018 within each age group, and more pronounced among young adults.

### Source of HIV test

**Fig. 3:**
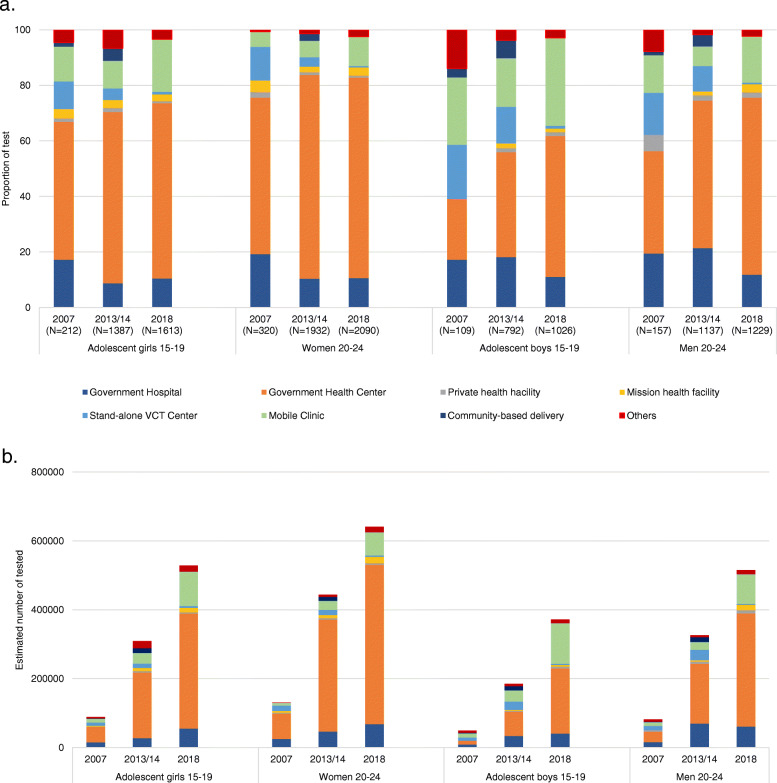
Changes in the source of reception of HIV tests between 2013 and 14 and 2018, for tests conducted in the last 12 months. **a**. The proportion of tests conducted through each source, **b**. estimated number of youths tested through each source of testing

Figure 3a shows the proportions of HIV tests received through each testing source between 2007 and 2018. Government health centres (GHC) accounted for more than half of HIV tests for adolescent girls and young women in 2007, and their proportion increased over time, with more than 60% in each age group getting tested at a GHC in 2018. GHCs accounted for a much smaller percentage among men in 2007 but increased considerably in 2013, and by 2018 became the source for more than 50% of HIV tests among men. Moreover, the percentage getting tested in mobile clinics (MC) almost doubled in all four groups between 2013 and 2018, reaching 32% among adolescent boys. The increase in the percentage of youth tested in either GHCs or MCs masks considerable increases in the absolute number of tests performed through these sources, due to the overall increase in the percentage of youth, as shown in Fig. 3b. For GHCs the estimated number of tests performed for youth increased substantially from roughly 163,000 in 2007 to 1.3 million in 2018. For MC sources, the overall estimated number of tests reached approximately 400,000 in 2018 compared to 41,000 in 2007.

### Determinants of HIV testing uptake in the 2018 DHS

Characteristics of the target population in the 2018 DHS dataset are shown in Table 1.

**Table 1:** Respondent characteristics of young women (*N* = 5799) and men (*N* = 4846), Zambia 2018 DHS

Characteristics	Women			Men		
	N	Percent (%)	95% CI	N	Percent (%)	95% CI
**Age**						
15–19	3112	52.3	[50.8–53.9]	2852	57.8	[55.9–59.3]
20–24	2687	47.7	[46.1–49.2]	1994	42.2	[40.4–44.1]
**Education level**						
No education	237	3.6	[2.9–4.4]	133	3.0	[2.3–4.0]
Primary	2313	39.0	[37.0–41.0]	1933	38.2	[36.0–40.4]
Secondary	3118	54.8	[52.8–56.8]	2666	55.9	[53.9–57.8]
Higher	131	2.6	[1.9–3.5]	114	2.9	[2.1–3.9]
**Household wealth quintile**						
Poorest	1186	17.9	[16.4–19.6]	849	15.1	[13.6–16.8]
Poorer	1132	17.5	[16.0–19.2]	961	17.3	[15.6–19.0]
Middle	1153	18.2	[16.5–20.1]	1073	20.8	[18.9–22.8]
Richer	1103	22.3	[19.5–25.4]	936	22.8	[19.2–26.7]
Richest	1225	24.1	[21.2–27.3]	1027	24.0	[20.8–27.7]
**Relationship status**						
Never in union	3681	63.1	[60.8–65.3]	4296	89.1	[87.8–90.3]
Currently in union	1879	32.7	[30.5–35.0]	514	10.2	[9.1–11.4]
Formerly in union	239	4.2	[3.6–4.9]	36	0.7	[0.5–1.0]
**Residence**						
Urban	2335	46.0	[43.1–48.9]	1812	44.1	[40.7–47.4]
Rural	3464	54.1	[51.2–57.0]	3034	56.0	[52.6–59.3]
**Region**						
Central	612	8.9	[7.9–9.9]	533	8.9	[7.9–10.1]
Copperbelt	683	16.2	[13.8–19.0]	558	15.3	[13.3–17.5]
Eastern	661	12.0	[10.6–13.5]	574	13.1	[11.7–14.7]
Luapula	599	7.8	[6.8–9.0]	530	8.1	[7.1–9.4]
Lusaka	688	18.6	[16.3–21.1]	547	17.5	[15.2–20.2]
Muchinga	501	5.6	[4.8–6.7]	435	5.6	[4.9–6.3]
Northern	542	8.0	[7.0–9.1]	422	7.6	[6.4–9.1]
North western	485	5.9	[4.7–7.5]	391	5.3	[4.4–6.5]
Southern	572	11.1	[9.0–13.7]	489	12.8	[9.5–17.2]
Western	456	5.9	[5.1–6.8]	367	5.6	[4.7–6.6]
**Ever had sex**						
No	1741	30.2	[28.4–32.0]	1732	36.8	[34.9–38.7]
Yes	4058	69.9	[68.0–71.7]	3114	63.2	[61.3–65.1]
**Age at first sexual intercourse**^b^						
Before 16 years	1740	41.8	[39.7–43.8]	1425	44.1	[41.7–46.4]
At 16 years and above	2318	58.3	[56.2–60.3]	1689	55.9	[53.6–58.3]
**Number of sexual partners in the last 12 months**						
None/never had sex	2354	40.2	[38.2–42.3]	2384	50.3	[48.3–52.3]
One	3350	58.1	[56.1–60.1]	1934	39.1	[37.2–41.1]
Two or more	95	1.7	[1.3–2.1]	528	10.6	[9.4–11.8]
**Condom used during last sexual intercourse**^b^						
No	3429	84.1	[82.5–85.6]	2119	67.4	[65.2–69.5]
Yes	629	15.9	[14.4–17.5]	995	32.6	[30.5–34.8]
**Heard about STI**						
No	115	1.8	[1.5–2.3]	43	0.8	[0.6–1.2]
Yes	5684	98.2	[97.7–98.5]	4803	99.2	[98.8–99.4]
**History of STI**						
No	5735	99.0	[98.6–99.3]	4692	97.0	[96.3–97.6]
Yes	64	1.0	[0.7–1.4]	154	3.0	[2.4–3.7]
**Heard about AIDS**						
No	189	3.0	[2.5–3.7]	76	1.5	[1.2–2.0]
Yes	5610	97.0	[96.3–97.5]	4770	98.5	[98.0–98.9]
**Reported stigma**						
No	888	15.5	[14.1–17.0]	517	9.3	[8.3–10.5]
Yes	4048	69.6	[67.7–71.3]	3765	79.4	[77.7–80.9]
Don’t know	863	14.9	[13.7–16.3]	564	11.3	[10.1–12.7]
**Discriminatory attitudes**						
No	3966	69.5	[67.9–71.1]	3401	70.1	[65.3–71.9]
Yes	1833	30.5	[28.9–32.1]	1445	29.9	[28.1–31.8]
**Comprehensive knowledge of HIV**						
No	3269	55.2	[53.1–57.3]	2802	57.7	[55.8–59.6]
Yes	2530	44.8	[42.7–47.0]	2044	42.3	[40.4–44.2]
**Knowledge of a place to get HIV test**^a^						
No	417	6.8	[6.0–7.7]	389	7.9	[6.9–8.9]
Yes	5382	93.2	[92.3–94.0]	4457	92.1	[91.1–93.1]
**HIV Self testing**						
Never heard of HIV self-test Kit	4908	85.4	[83.7–87.0]	3926	80.3	[78.4–82.1]
Has tested with HIV self-test Kit	105	2.6	[2.0–3.5]	89	2.5	[1.8–3.4]
Knows self-test kits but never tested with	597	12.0	[10.8–13.2]	755	17.2	[15.7–18.9]
**Circumcised**						
*No*				2952	62.6	[60.4–64.7]
Yes				1894	37.4	[35.3–39.6]
**Exposure to television**						
Not at all	3582	56.7	[53.8–59.6]	2424	45.6	[42.9–48.3]
Less than once a week	341	6.0	[5.2–6.9]	565	11.9	[9.9–14.3]
At least once a week	1876	37.3	[34.6–40.1]	1857	42.5	[39.8–45.3]
**Exposure to radio**						
Not at all	3307	55.2	[53.1–57.3]	1766	34.6	[32.5–36.8]
Less than once a week	721	13.6	[12.4–14.8]	697	15.0	[13.6–16.6]
At least once a week	1771	31.2	[29.3–33.1]	2383	50.4	[48.0–52.7]
**Use of internet**						
Not at all	5123	87.3	[85.6–88.7]	3709	73.8	[71.5–76.1]
Less than once a week	118	2.1	[1.7–2.6]	234	5.3	[4.5–6.3]
At least once a week	218	4.1	[3.4–4.9]	457	9.6	[8.4–11.0]
Almost everyday	340	6.5	[5.5–7.7]	446	11.2	[9.5–13.2]
**Owns a mobile phone**						
No	3471	56.8	[54.6–58.9]	2313	46.1	[43.8–48.5]
Yes	2328	43.2	[41.1–45.4]	2533	53.9	[51.6–56.2]
**HIV test in the last 12 months and received the result**						
No	2096	34.8	[33.1–36.6]	2591	51.2	[48.4–54.0]
Yes	3703	65.2	[63.4–67.0]	2255	48.8	[46.0–51.6]

^a^Comprehensive knowledge is a composite of 4 questions to assess respondent understanding of HIV transmission (whether people reduce their chances of getting the AIDS virus by having just one uninfected sex partner who has no other partners and if a healthy-looking person can have AIDS) and prevention (whether a person can get the AIDS virus from mosquito bites and if a person can get the AIDS virus by sharing food with a person who has AIDS)

^b^The frequency and percentage for the age at first sexual intercourse and use of condom are restricted to respondents who report prior sexual intercourse

In the multivariate models, the ages 20–24 were commonly associated with high odds of HIV testing among both non-pregnant women (adjusted odds ratio = 1.74, 95%CI:1.40–2.15) and men (aOR = 1.55, 95%CI:1.30–1.84) (Tables 2 and 3). Among women the odds of testing increased with the level of education attained (aOR = 6.97; 95%CI:2.82–17.19, for higher education compared to no education). Among men high HIV testing uptake was mainly predicted by being circumcised (aOR = 1.57; 95%CI:1.32–1.88) and currently being in a union (aOR = 2.46, 95%CI:1.85–3.28, compared to never in union).

**Table 2:** Determinants of HIV testing uptake among non-pregnant women ages 15–24, Zambia 2018 DHS (*N* = 4198)

Respondent characteristics	N	HTC coverage (%)	Crude OR(95% CI)	***P***-value	Adjusted OR(95% CI)	***P***-value
**Age**				< 0.001		< 0.001
15–19	2484	45.8	1		1	
20–24	1714	74.4	3.44 [2.98–3.98]		1.74 [1.40–2.15]	
**Education level**				< 0.001		< 0.001
No education	145	22.9	1		1	
Primary	1521	47.0	3.00 [1.89–4.75]		3.14 [1.94–5.08]	
Secondary	2418	65.5	6.41 [3.98–10.32]		5.52 [3.30–9.25]	
Higher	114	77.3	11.49 [5.14–25.68]		6.97 [2.82–17.19]	
**Household wealth quintile**				< 0.001		0.04
Poorest	719	48.5	1		1	
Poorer	730	50.9	1.10 [0.88–1.38]		0.99 [0.76–1.29]	
Middle	790	56.3	1.37 [1.08–1.74]		1.02 [0.76–1.37]	
Richer	858	68.4	2.30 [1.74–3.04]		1.41 [0.96–2.09]	
Richest	1101	59.3	1.55 [1.23–1.96]		0.90 [0.59–1.38]	
**Relationship status**				< 0.001		0.04
Never in union	3185	53.0	1		1	
Currently in union	850	73.5	2.46 [2.03–2.99]		1.33 [0.99–1.79]	
Formerly in union	163	73.1	2.42 [1.58–3.68]		1.70 [1.04–2.78]	
**Residence**				< 0.001		0.23
Urban	1915	63.3	1		1	
Rural	2283	52.5	0.64 [0.54–0.76]		0.85 [0.65–1.11]	
**Region**				< 0.001		< 0.001
Central	445	57.3	0.85 [0.62–1.16]		1.10 [0.78–1.54]	
Copperbelt	549	61.3	1		1	
Eastern	419	53.4	0.72 [0.52–1.01]		1.11 [0.76–1.62]	
Luapula	414	41.5	0.45 [0.34–0.60]		0.66 [0.48–0.90]	
Lusaka	560	63.8	1.11 [0.82–1.51]		1.00 [0.71–1.40]	
Muchinga	363	41.7	0.45 [0.31–0.67]		0.68 [0.47–0.99]	
Northern	371	46.1	0.54 [0.37–0.79]		0.90 [0.58–1.41]	
North western	362	58.8	0.90 [0.62–1.32]		1.07 [0.73–1.58]	
Southern	406	67.1	1.29 [0.91–1.82]		1.44 [0.98–2.11]	
Western	309	73.0	1.71 [1.18–2.47]		2.48 [1.63–3.77]	
**Number of sexual partners**				< 0.001		< 0.001
None	2255	45.5	1		1	
One	1861	72.6	3.17 [2.72–3.70]		2.13 [1.74–2.61]	
Two or more	82	67.2	2.45 [1.37–4.40]		1.61 [0.79–3.31]	
**History of STI**				0.006		0.16
No	4161	57.9	1		1	
Yes	37	87.1	4.90 [1.60–15.03]		2.63 [0.68–10.16]	
**Reported stigma**				< 0.001		< 0.001
No	607	63.2	1.08 [0.87–1.34]		1.26 [0.98–1.61]	
Yes	2937	61.3	1		1	
Don’t know	654	39.0	0.40 [0.32–0.52]		0.55 [0.42–0.72]	
**Discriminatory attitudes**				0.01		0.12
No	2957	59.7	1		1	
Yes	1241	54.1	0.80 [0.67–0.95]		1.16 [0.96–1.39]	
**Comprehensive knowledge of HIV**				< 0.001		0.18
No	2311	53.1	1		1	
Yes	1887	63.8	1.56 [1.32–1.83]		1.13 [0.95–1.35]	
**Exposure to television**				0.007		0.17
Not at all	2368	55.8	1		1	
Less than once a week	257	53.6	0.91 [0.69–1.22]		0.72 [0.51–1.01]	
At least once a week	1573	61.5	1.27 [1.07–1.50]		0.88 [0.67–1.16]	
**Exposure to radio**				0.003		0.21
Not at all	2311	56.1	1		1	
Less than once a week	537	55.6	0.98 [0.76–1.25]		0.91 [0.69–1.18]	
At least once a week	1350	62.3	1.29 [1.09–1.53]		1.13 [0.92–1.40]	
**Owns a mobile phone**				< 0.001		< 0.001
No	2400	45.5	1		1	
Yes	1798	72.6	3.17 [2.71–3.70]		1.88 [1.52–2.33]	
**Use of internet**				< 0.001		0.24
Not at all	3609	56.0	1		1	
Less than once a week	100	55.0	0.96 [0.60–1.54]		0.60 [0.34–1.05]	
At least once a week	186	73.1	2.14 [1.42–3.21]		1.26 [0.80–1.98]	
Almost everyday	303	72.2	2.04 [1.55–2.69]		0.98 [0.69–1.40]

**Table 3:** Determinants of HIV testing uptake among men ages 15–24, Zambia 2018 (*N* = 4846)

Respondent characteristics	N	HTC coverage (%)	Crude OR(95% CI)	***P*** value	Adjusted OR(95% CI)	***P*** value
**Age**				< 0.001		< 0.001
15–19	2852	37.5	1		1	
20–24	1994	64.2	2.98 [2.58–3.45]		1.55 [1.30–1.84]	
**Education level**				< 0.001		< 0.001
No education	133	37.8	1		1	
Primary	1933	37.1	0.97 [0.59–1.61]		1.02 [0.68–1.51]	
Secondary	2666	56.4	2.13 [1.33–3.40]		1.51 [1.01–2.26]	
Higher	114	68.8	3.63 [1.98–6.65]		1.41 [0.74–2.71]	
**Household wealth quintile**				0.001		< 0.001
Poorest	849	39.8	1		1	
Poorer	961	43.4	1.16 [0.93–1.44]		1.23 [0.95–1.59]	
Middle	1073	49.7	1.49 [1.20–1.86]		1.36 [1.03–1.79]	
Richer	936	56.8	1.99 [1.38–2.87]		1.37 [0.99–1.90]	
Richest	1027	50.0	1.52 [1.21–1.90]		0.78 [0.53–1.15]	
**Relationship status**				< 0.001		< 0.001
Never in union	4296	45.8	1		1	
Currently in union	514	74.0	3.37 [2.60–4.37]		2.46 [1.85–3.28]	
Formerly in union	36	65.1	2.21 [0.97–5.04]		1.78 [0.74–4.27]	
**Residence**				< 0.001		0.06
urban	1812	54.6	1		1	
rural	3034	44.2	0.66 [0.52–0.83]		0.72 [0.52–1.01]	
**Region**				< 0.001		0.002
Central	533	52.1	1.13 [0.84–1.51]		1.44 [1.00–2.06]	
Copperbelt	558	49.2	1		1	
Eastern	574	49.1	1.00 [0.74–1.34]		1.41 [0.97–2.05]	
Luapula	530	39.8	0.68 [0.50–0.92]		0.96 [0.67–1.38]	
Lusaka	547	52.4	1.12 [0.84–1.54]		1.10 [0.79–1.53]	
Muchinga	435	33.3	0.51 [0.35–0.76]		0.76 [0.49–1.20]	
Northern	422	41.6	0.73 [0.53–1.03]		1.12 [0.77–1.64]	
North western	391	44.8	0.84 [0.60–1.17]		0.75 [0.51–1.10]	
Southern	489	56.4	1.34 [0.70–2.54]		1.39 [0.75–2.58]	
Western	367	55.1	1.27 [0.93–1.73]		1.45 [0.98–2.13]	
**Ever had sex**				< 0.001		0.14
No	1732	34.0	1		1	
Yes	3114	57.4	2.62 [2.27–3.01]		1.19 [0.95–1.50]	
**Number of sexual partners**				< 0.001		< 0.001
None	2384	37.1	1		1	
One	1934	61.2	2.67 [2.30–3.09]		1.79 [1.41–2.27]	
Two or more	528	58.4	2.37 [1.84–3.06]		1.38 [0.99–1.91]	
**History of STI**				0.26		
No	4692	48.6	1		–	
Yes	154	54.1	1.25 [0.85–1.82]		–	
**Circumcised**				< 0.001		< 0.001
No	2952	43.8	1		1	
Yes	1894	57.2	1.72 [1.46–2.02]		1.58 [1.32–1.89]	
**Reported stigma**				< 0.001		< 0.001
No	517	40.6	0.64 [0.50–0.81]		0.65 [0.50–0.84]	
Yes	3765	51.8	1		1	
Don’t know	564	34.3	0.49 [0.40–0.59]		0.61 [0.49–74]	
**Discriminatory attitudes**				< 0.001		0.02
No	3401	52.3	1		1	
Yes	1445	29.8	0.63 [0.53–0.73]		0.80 [0.67–0.97]	
**Comprehensive knowledge of HIV**				< 0.001		0.14
No	2802	44.8	1		1	
Yes	2044	54.2	1.46 [1.28–1.66]		1.13 [0.96–1.33]	
**Owns a mobile phone**				< 0.001		0.002
no	2313	36.8			1	
yes	2533	59.0	2.47 [2.10–2.89]		1.32 [1.11–1.57]	
**Use of internet**				< 0.001		< 0.001
Not at all	3709	43.5	1		1	
Less than once a week	234	57.8	1.78 [1.32–2.39]		1.41 [1.02–1.94]	
At least once a week	457	63.0	2.21 [1.69–2.87]		1.53 [1.13–2.08]	
Almost everyday	446	66.9	2.62 [2.04–3.37]		1.76 [1.32–2.35]	
**Exposure to television**				< 0.001		0.07
Not at all	2424	42.7	1		1	
Less than once a week	565	57.9	1.85 [1.30–2.61]		1.38 [1.05–1.82]	
At least once a week	1857	52.7	1.50 [1.29–1.73]		1.08 [0.85–1.38]	
**Exposure to radio**				< 0.001		0.04
Not at all	1766	43.9	1		1	
Less than once a week	697	48.6	1.21 [1.00–1.47]		0.86 [0.69–1.07]	
At least once a week	2383	52.2	1.40 [1.22–1.59]		1.12 [0.94–1.34]	

Among men and non-pregnant women who reported previous sexual experience, condom use at last intercourse was not associated with HIV testing uptake among women but was a predictor of testing among men (aOR = 1.64, 95%CI: 1.32–2.04) (Tables S2 and S3 in SM). Other predictors of testing were like the main models, except for not reporting HIV-related stigma manifestation which was associated with higher odds of testing among non-pregnant sexually active women (aOR = 1.59, 95%CI:1.14–2.21, compared to those reporting about stigma) (Tables S2); while having a discriminatory attitude was no longer a predictor for among men.

### Inequity in HIV testing uptake

**Fig. 4:**
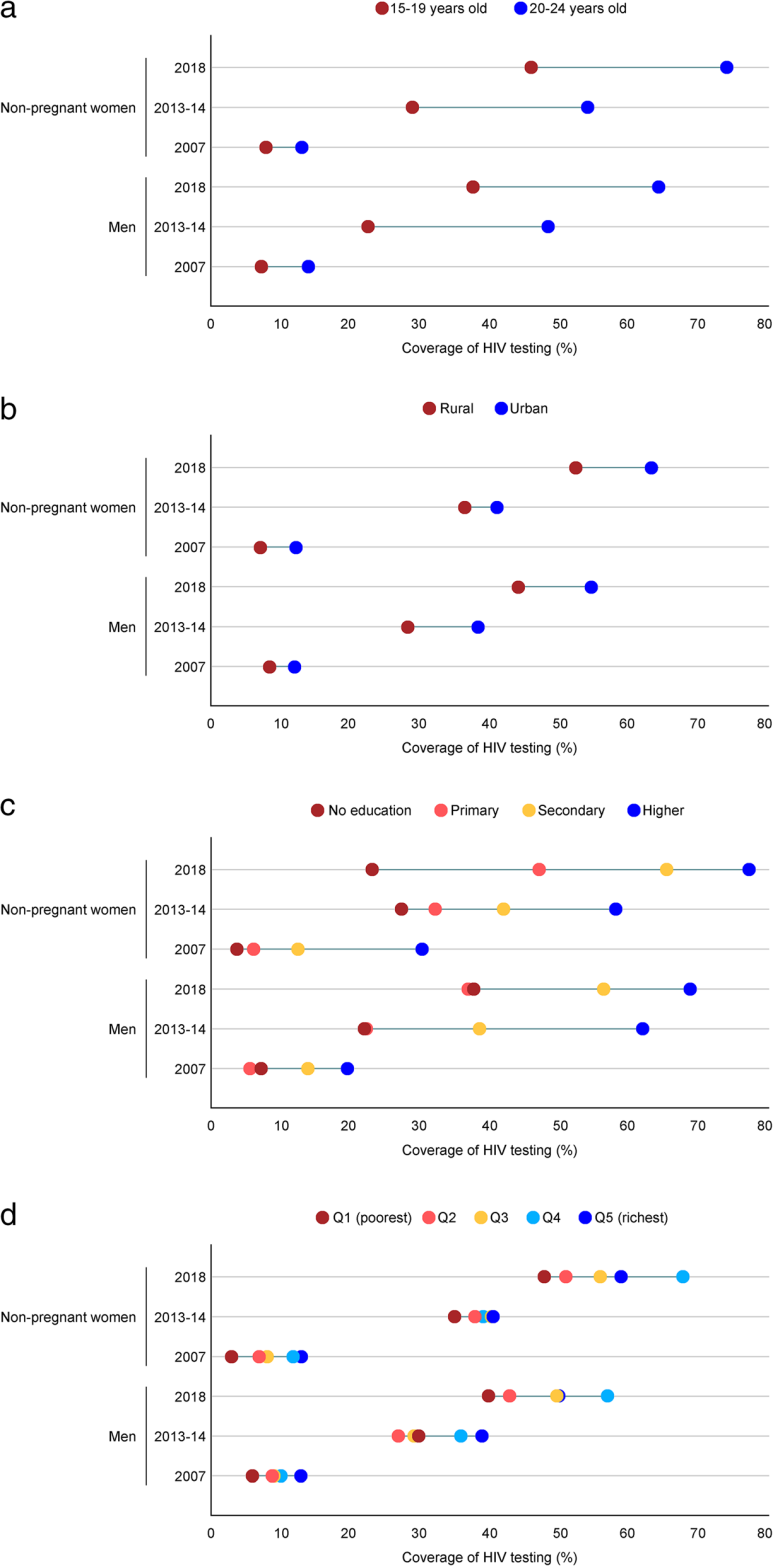
Changes in inequities of testing uptake among men and young non-pregnant women tested and receiving the results in the past 12 months, between 2007 and 2018. **a**. age-based inequalities, **b**. residence-based inequalities, **c**. education-based inequalities, **d**. wealth-based inequalities

**Fig. 5:**
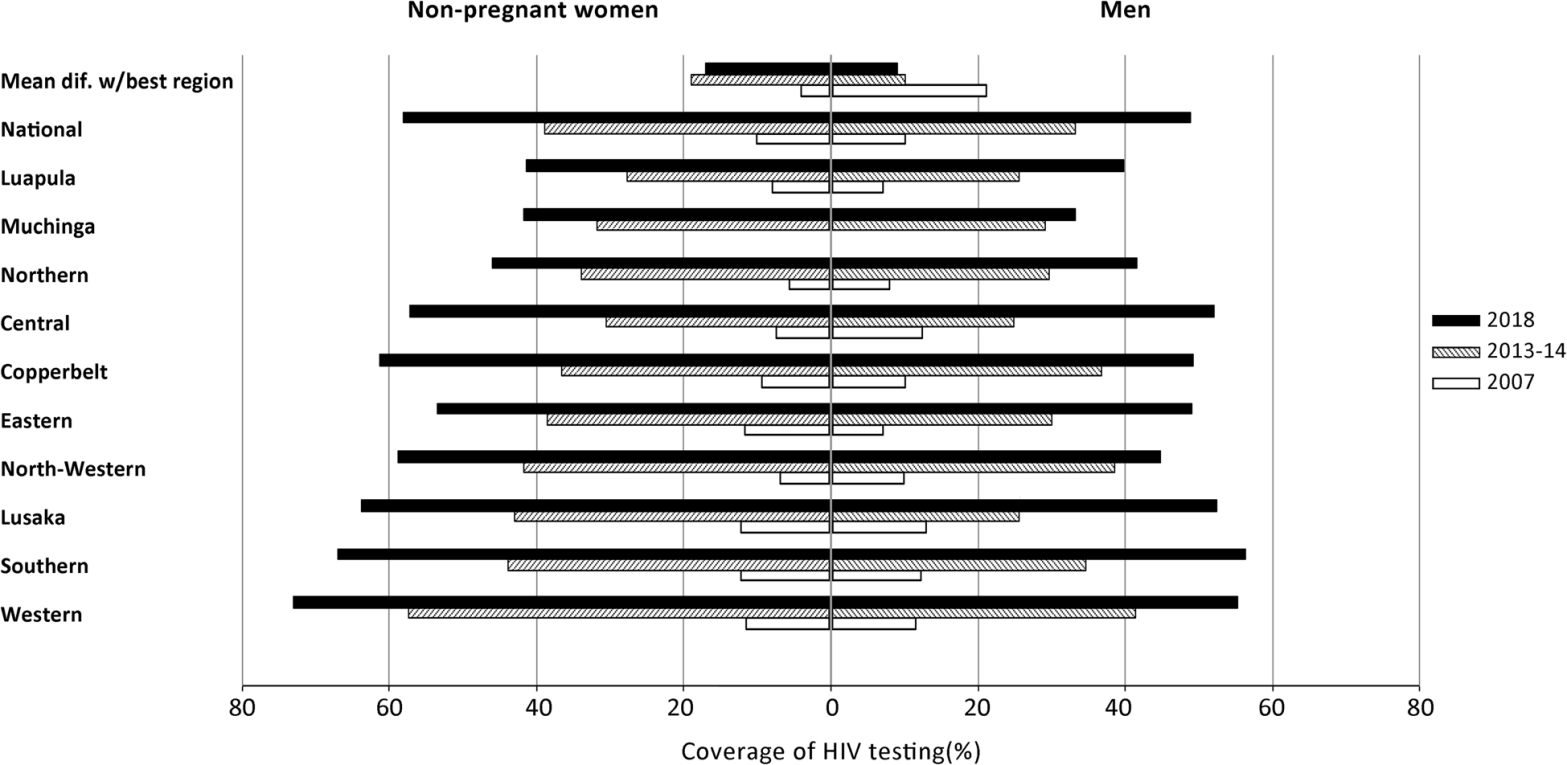
Changes in testing coverage among men and non-pregnant women tested and receiving results in the past 12 months, between 2007 and 2018. Region-based inequalities

We analysed inequities in HIV testing uptake for a test taken and results received in the last 12 months for age, residence, education level, household wealth, and regions at each time point among men and young non-pregnant women. The results suggested an overall improvement in testing coverage between 2007 and 2018 in each sub-group for all inequality qualifiers (Fig. 4). However, by 2018 the absolute inequity in coverage widened between sub-groups for both genders. Less well covered were those age 15–19 years-old, living in rural areas, less educated, and the poorest (Figs. 4a-d). Education-based inequity was more considerable by 2018 among women (54% absolute difference between no education and higher education) than men (31%) (Fig. 4c). Regarding regions, by 2018 the disparities in testing coverage across regions increased among women (increase in the mean difference from the best-covered region from 4% in 2007 to 17% in 2018) (Fig. 5). Among men, however, an opposite trend was observed between 2007 and 2018; specifically, a reduction in the mean difference from the best-covered region from 21% in 2007 to 9% in 2018.

## Discussion

This study analyses in-depth the uptake of HTC services among youth in Zambia, using nationally representative population-based survey data. We demonstrate an increase in uptake of HTC between 2007 and 2018, from 45 to 92% among pregnant women, 10 to 58% among non-pregnant women, and from 10 to 49% among men. Government health centres became the primary source of HIV testing by 2018, performing around 60% of all tests among youth. The percentage of tests delivered through mobile clinics almost doubled in all groups between 2013 and 2018 and accounted for one-third of all tests among adolescent boys. Multivariate analysis conducted for men and non-pregnant women showed higher odds of testing among young adults than adolescents (aOR = 1.55 among men and aOR = 1.74 among women). Circumcision and in a union were associated with higher odds of testing among men, whereas higher education and not reporting HIV-related stigma were predictors of testing among non-pregnant sexually active women. Inequity analyses mainly found an improvement in testing coverage in each sub-group of all inequality qualifiers by 2018, although the absolute difference in coverage was widened between the sub-groups for both genders. Education-based inequity was substantially increased among women than men by 2018.

The trend observed in this study for HTC uptake among youth demonstrates a considerable improvement over time in Zambia. Among pregnant women, the achievement could be related to the integration into ANC services since 2005 of a program of prevention of mother to child transmission of HIV (PMTCT), intensively promoted among pregnant women to ensure almost universal access to HTC around 2013 [40, 41]. The great level of attendance to ANC services among pregnant Zambian women was reported by the 2007 DHS (97% of women with at least one ANC visit) and maintained in 2013–14 and 2018 (roughly 98% for both reports); and was likely a contributing factor for inclusion of young women regardless of their age [7, 24, 42].

Regarding men, the promotion of couple HIV counselling and testing (CHCT) among partners of women attending ANC might be a factor to consider, especially considering that multivariate analysis in our study showed a high odds of testing among men in a union [43–45]. In our results the voluntary medical male circumcision (VMMC) as part of the main predictors of HIV testing among young men suggests a potential contribution of the VMMC campaigns launched in 2012 in Zambia. These campaigns reached over 400,000 men by 2013, and included HIV testing and other preventive services in addition to circumcision [46, 47]. Its scale-up in 2016, mainly through the mobile clinics, might explain the increase in the proportion of this source of delivery as reflected in our results for adolescent boys in 2018 (32% of testing through MC). The increasing proportion of HIV tests offered through MC observed in the study reflects an attempt of the Zambian government to reach underserved and hard-to-reach youth. In addition to the latter, other community-based strategies that are specific to youth should be explored given their promising results, such as adolescent-focused case finding implemented in Kenya and home-based HTC [48, 49]. HIV self-testing (HST) is also part of interventions in Zambia and has shown some acceptance and the potential to improve access to HIV testing [50–53]. However, our study found that HST was unknown to most youth (85 and 80% among women and men, respectively). Its promotion, together with other community-based approaches, is to be encouraged given their potential to increase testing coverage, overcome stigma barriers, and contribute to reducing risky sexual behaviour [54–58]. Concerns regarding their linkage to care for HIV positive cases should be adequately addressed if chosen to be implemented at a large scale.

The positive changes in testing uptake highlighted above among men and non-pregnant women have also been accompanied by a constant gap in the trend of HIV testing coverage between genders, with men being generally less well covered than women. Similar differences among youth were reported in Nigeria, Mozambique, and Uganda [59, 60]. The persistence of higher testing rates among non-pregnant women compared to men may be due to their higher demand of HTC services, caused by a greater perception of HIV risk resulting from their vulnerability and frequent exposures to sexual intercourse with older partners with whom they may have less control over condom use [10, 61]. Women of reproductive age are also generally reported to use primary healthcare more often than men, either for themselves or for their children [62, 63]. As a result, non-pregnant women remain more likely to be suggested an HIV test whenever they interact with health services as part of provider-initiated counselling and testing (PICT), which is widely implemented in government health facilities in Zambia [26, 46, 64]. Moreover, it is possible that existing interventions that target youth, such as youth-friendly services (YFS), might be much more women-specific [65, 66]. Indeed, it has been shown that norms related to masculinity bring men to consider sexual health as a woman’s domain, and therefore believe that it would be inappropriate for them [67]. A recent scoping review focusing on the sub-Sahara African (SSA) region highlighted several other barriers to uptake of HTC among men that might be important to consider even for youth [68, 69]. Among the most common, we find poor knowledge of HIV, fear of testing positive, lack of confidentiality, and other aspects related to the quality of services. Therefore, increasing uptake of testing among young men will require the implementation of interventions that are young men-driven, needs-based, and beneficiary responsive, including implementation of decentralised service delivery models that capture young men in their safe spaces.

Our results showed adolescent girls (non-pregnant) and boys having a lower HIV testing uptake by 2018 (46 and 38%, respectively), compared to young adults. The persistence of this age-based gap in the trend analysis was observed in both multivariate and inequity analysis among both non-pregnant women and men. The proportions achieved in testing coverage among adolescents in 2018 are still far from the testing targets set by the Zambian Ministry of Health for this year (70 and 50% for adolescent girls and boys, respectively) [10]. A recent study in Zambia and several other countries from the SSA region have also reported lower odds of testing among adolescents [21, 33, 59, 60]. Most supported the fact that older age is likely to confer more sexual experience and better knowledge of HIV, which may accordingly improve the perception of the risk and affect the need for HIV testing [21, 60, 70]. Other barriers specific to adolescents include the legal age of consent to HIV testing, stigma, and sanctioning of sexual activity in adolescents; and are important to be highlighted to ensure that they are targets of future interventions that aim to improve coverage of testing among adolescents in Zambia [22, 71, 72]. The ongoing mobilization in Zambia to revise the legal age of consent, currently at 16 years old, needs to be further supported and accelerated [10, 65]. Lowering the age of consent below 16 years old is associated with increased testing for adolescents (11% increase in national testing coverage, 95%CI:7.2–14.8%), as suggested in a systematic review that included several high burden countries [73, 74].

Of the other determinants analysed, having reported HIV-related stigma was associated with lower odds of HTC among non-pregnant sexually active women. The negative impact of stigma has been noted by several other authors and remains a challenge for any HIV program [75, 76]. However, it should be recognized that the scaling up of HIV prevention and treatment services, especially in a universal ‘test and treat’ approach, could help reduce HIV-related stigma in the community through several pathways and improve access to these services [77, 78]. We also found strong evidence of higher odds of HIV testing among the most educated women, consistent with other studies on youth in the SSA region [21, 33, 59]. The education-based inequity widened in the last survey, mostly among non-pregnant women, indicating the need to reach the least educated youths. Other sub-groups of disadvantaged young people who were identified from the inequity analysis require continual attention to ensure improvement of the testing coverage among youth in Zambia.

The results from this study suggest some critical actions from programme implementers and researchers to ensure better access to HTC for youth in Zambia. These include the scaling-up of mobile testing and strengthening of alternative community-based approaches such as HIV self-testing, which has shown some acceptance and potential to clients who are less easy to reach through the government health facilities. The development of gender-sensitive HTC services and less coercive strategies to sustain the gain in testing uptake among men in a union are also important to consider. Finally, the warning about barriers associated with the access to sexual health and HIV services through YFS in a recent study from Brazil [79], and the scarcity of evidence supporting the progress made since their introduction in Zambia, suggest that more research will help to demonstrate their contribution and yield.

## Conclusion

Overall, the improvement observed in HTC among Zambian youth is encouraging, with 65% of women and 49% of men knowing their status, although it is still far from the 95% goal envisioned by the UNAIDS in 2030. Therefore, renewed efforts are needed to close the gaps observed among men in general, non-pregnant and less educated adolescent girls, and young women. Sustaining the gains obtained from existing HTC services by addressing barriers such as stigma and offering gender and adolescent-sensitive services is required, in addition to the scaling up of most effective community-based testing approaches. Despite the hope stemming from the recent mobilization to prioritize adolescent health in the country, much attention should be invested in rigorously tracking progress in access to HIV prevention and care services to ensure the reach, effectiveness, and sustainability of implemented strategies, as well as headway toward ensuring that youth live free of HIV and can contribute to the prosperity of the country.

## Supplementary Information

**Additional file 1: Table S1.** Details on variables used for the analysis; **Table S2**. Determinants of HIV testing uptake among sexually active non-pregnant young women aged 15–24, Zambia 2018 DHS (*N* = 2457); and **Table S3**. Determinants of HIV testing uptake among sexually active young men aged 15–24, Zambia DHS 2018 (*N* = 3114).

## Data Availability

Required permission was obtained from the DHS programme to access the data analysed for this study. All data and DHS-related materials used are available from the website: https://dhsprogram.com/.

## Abbreviations

AGAPE: Adolescent girls accessing prevention and education
AGYW: Adolescent girls and young women
AIDS: Acquired immune deficiency syndrome
ANC: Antenatal care
aOR: Adjusted odds ratio
CI: Confidence interval
CHCT: Couple HIV counselling and testing
cOR: Crude odds ratio
DHS: Demographic and health survey
DREAMS: Determined, resilient, empowered, AIDS-free, mentored, and safe
GFATM: Global fund to fight tuberculosis, HIV/AIDS and malaria
GHC: Government health centres
HIV: Human immunodeficiency virus
HTC: HIV testing and counselling
HST: HIV self-testing
LMIC: Low- and middle-income countries
NASF: National AIDS strategic framework
MC: Mobile clinic
PEPFAR: United States president’s emergency plan for AIDS relief
PICT: Provider-initiated counselling and testing
SM: Supplementary materials
SSA: Sub-Saharan Africa
STI: Sexually transmitted infection
UNAIDS: Joint United Nations programme on HIV/AIDS
UNICEF: United Nations children’s fund
VCT: Voluntary counselling and testing
VMMC: Voluntary medical male circumcision
YFS: Youth-friendly services
