# Supplementary material for: Changes over time in HIV testing and counselling uptake and associated factors among youth in Zambia: a cross-sectional analysis of demographic and health surveys from 2007 to 2018
Source: BMC Public Health. 2021 Mar 6;21:456. doi: 10.1186/s12889-021-10472-x (PMC7937241; doi:10.1186/s12889-021-10472-x)
Supplement: Supplementary file 1 — Additional file 1: Table S1. Details on variables used for the analysis; Table S2. Determinants of HIV testing uptake among sexually active non-pregnant young women aged 15–24, Zambia 2018 DHS (N = 2457); and Table S3. Determinants of HIV testing uptake among sexually active young men aged 15–24, Zambia DHS 2018 (N = 3114). [file 12889_2021_10472_MOESM1_ESM.docx]

**TITLE**: Changes over time in HIV testing and counselling uptake and associated factors among youth in Zambia: A cross-sectional analysis of Demographic and Health Surveys from 2007 to 2018.

**AUTHORS:**

Aimé Bitakuya Heri^1^

Francesca L Cavallaro^2^

Nurilign Ahmed^3^

Maurice Mubuyaeta Musheke^4^

Mitsuaki Matsui^1^

**INSTITUTIONS:**

1. Department of Global Health, Nagasaki University School of Tropical Medicine and Global Health
   Sakamoto 1-12-4, Nagasaki 852-8523, Japan
2. Great Ormond Street Institute of Child Health, University College London
   Gower Street, London, WC1E 6BT, United Kingdom
3. Faculty of Public Health and Policy, London School of Hygiene and Tropical Medicine
   Keppel Street, London WC1E 7HT, United Kingdom.
4. Centre for Infectious Disease Research in Zambia
   Plot # 34620, Off Alick Nkhata Road, Lusaka, Zambia

Corresponding author:

Mitsuaki Matsui, e-mail mmatsui@nagasaki-u.ac.jp

Table S1: Details on variables used for the analysis.

| **Variables** | **Response categories** |
| --- | --- |
| **Determinants of HIV testing for each Anderson' factors** | |
| 1. **Predisposing factors** |  |
| Age | Yes/No |
| Marital status | Never in union/ Currently in union/Formally in union |
| Education | None/Primary/Secondary/ Higher |
| 1. **Enabling factors** |  |
| Household wealth quintile | Poorest/Poorer/Middle/Richer/Richest |
| Residence | Urban/Rural |
| Regions | Central, Copperbelt, Eastern, Luapula, Lusaka, Muchinga, Northern, North western, Southern, and Western. |
| Exposure to TV | Not at all/Less than once a week/At least once a week |
| Exposure to radio | Not at all/Less than once a week/At least once a week |
| Use of the Internet | Never/Past 12 months/More than 12 months |
| Ownership of a mobile phone | Yes/No |
| Knowledge of a place to get an HIV test | Yes/No/not heard about HIV |
| Circumcised | Yes/No |
| 1. **Need factors** |  |
| Ever heard of AIDS | Yes/No |
| Comprehensive knowledge about HIV | Yes/No  Yes, if all answers to the questions below are correct.  Which implies an affirmative answer for questions 1 and 4 and negative answers for questions 2 and 3 below:   1. People can reduce their chances of getting the AIDS virus by having just one uninfected sex partner who has no other partners 2. A person can get the AIDS virus from mosquito bites 3. A person can get the AIDS virus by sharing food with a person who has AIDS 4. Can a healthy-looking person have AIDS? |
| Ever had sex | Implies ever had a sexual intercourse  Yes/No |
| Number of sexual partners | Implies the number of sexual partner in the past 12 months  None ( + never had sex)/1/2 or More |
| Age at first sex | Before 16 years and at 16 and above |
| Condom use during last intercourse | Yes/No |
| Heard about STI | Yes/No |
| Reported STI | Implies reported STI in the past 12 months  Yes/No |
| Reported stigma | Implies reporting negative (individual- and population-level ) manifestations of HIV-related stigma.  Coded as Yes/No:  Yes, if an affirmative response to at least one statements below:   1. Think people talk badly about people living or suspected of living with HIV 2. Thinks that people hesitate to take an HIV test because they are afraid of how other people will react to a positive result 3. People living with HIV lose the respect of others 4. I would be ashamed if a family member has HIV |
| Discriminatory attitudes | Implies holding discriminatory/stigmatizing attitudes towards people living with HIV.  Coded as Yes/No:  Yes, if a negative response to at least one of the statements below:   1. Children with HIV should be allowed to go to school with children who do not have HIV 2. Would buy vegetable from a vendor with HIV |
| Sources of HIV test | |
| Government hospital | As defined in the DHS aHospitals |
| Government health centre | Including public health centre, family planning clinic and health post |
| Private health facility | For private hospital, clinic and private doctor |
| Mission health facility | Including all non-governmental hospitals and clinics |
| Stand-alone voluntary and testing clinic (VCT) centre | As defined in the DHS |
| Mobile clinic | Regrouping public and private ones |
| Community-based delivery | For both public and private ones and including delivery at home, workplace and prison |
| Others | Including pharmacy, as well as other private and public sources |

Table S2: Determinants of HIV testing uptake among sexually active non-pregnant young women aged 15-24, Zambia 2018 DHS (N=2457).

| **Repondent characteristics** | **N  (% coverage)** | **Crude OR (95% CI)** | **P-value** | **Adjusted OR (95% CI)** | **P-value** |
| --- | --- | --- | --- | --- | --- |
| **Age** |  |  | <0.001 |  | <0.001 |
| 15-19 | 940 (61.7) | 1 |  | 1 |  |
| 20-24 | 1517 (77.4) | 2.12 [1.72-2.62] |  | 1.75 [1.39-2.21] |  |
| **Education level** |  |  | <0.001 |  | <0.001 |
| No education | 96 (31.0) | 1 |  | 1 |  |
| Primary | 884 (63.7) | 3.92 [2.37-6.48] |  | 3.29 [1.84-5.85] |  |
| Secondary | 1396 (78.5) | 8.13 [4.86-13.62] |  | 4.38 [2.34-8.18] |  |
| Higher | 81 (82.8) | 10.72 [4.51-25.49] |  | 4.04 [1.42-11.51] |  |
| **Wealth Index** |  |  | <0.001 |  | 0.61 |
| Poorest | 483 (59.4) | 1 |  | 1 |  |
| Poorer | 466 (67.1) | 1.39 [1.03-1.87] |  | 1.09 [0.79-1.50] |  |
| Middle | 491 (70.7) | 1.64 [1.19-2.28] |  | 0.99 [0.68-1.43] |  |
| Richer | 518 (78.0) | 2.42 [1.72-3.41] |  | 1.06 [0.66-1.69] |  |
| Richest | 499 (78.3) | 2.46 [1.79-3.38] |  | 0.99 [0.57-1.75] |  |
| **Relationship status** |  |  | 0.42 |  |  |
| Never in union | 1444 (70.6) | 1 |  | - |  |
| Currently in union | 850 (73.5) | 1.16 [0.92-1.45] |  | - |  |
| Formerly in union | 163 (73.1) | 1.14 [0.74-1.74] |  | - |  |
| **Residence** |  |  | <0.001 |  | 0.35 |
| Urban | 1045 (78.4) | 1 |  | 1 |  |
| Rural | 1412 (65.6) | 0.53 [0.42-0.66] |  | 0.86 [0.62-1.19] |  |
| **Region** |  |  | <0.001 |  | <0.001 |
| Central | 241 (74.7) | 0.73 [0.47-1.14] |  | 1.09 [0.67-1.76] |  |
| Copperbelt | 265 (80.2) | 1 |  | 1 |  |
| Eastern | 276 (63.1) | 0.42 [0.27-0.65] |  | 0.83 [0.52-1.34] |  |
| Luapula | 221 (52.6) | 0.27 [0.19-0.39] |  | 0.42 [0.27-0.65] |  |
| Lusaka | 319 (78.3) | 0.89 [0.59-1.36] |  | 0.93 [0.60-1.45] |  |
| Muchinga | 187 (58.8) | 0.35 [0.21-0.60] |  | 0.61 [0.36-1.04] |  |
| Northern | 197 (61.3) | 0.39 [0.24-0.64] |  | 0.75 [0.43-1.29] |  |
| North western | 253 (72.4) | 0.67 [0.40-1.12] |  | 1.04 [0.61-1.79] |  |
| Southern | 267 (73.9) | 0.70 [0.44-1.13] |  | 1.00 [0.61-1.62] |  |
| Western | 231 (77.6) | 0.86 [0.53-1.37] |  | 1.66 [1.00-2.75] |  |
| **Age at first sex** |  |  | <0.001 |  | 0.25 |
| Befor 16 years | 1045 (66.0) | 1 |  | 1 |  |
| At 16 years and above | 1412 (75.4) | 1.55 [1.26-1.90] |  | 1.15 [0.91-1.46] |  |
| **Condom used** |  |  | 0.10 |  |  |
| No | 1942 (70.9) | 1 |  | - |  |
| Yes | 515 (75.1) | 1.24 [0.96-1.61] |  | - |  |
| **Number of sexual partners** |  |  | 0.32 |  |  |
| None | 514 (69.5) | 1 |  | - |  |
| One | 1861 (72.6) | 1.16 [0.92-1.47] |  | - |  |
| Two or more | 82 (71.8) | 0.90 [0.49-1.67] |  | - |  |
| **History of STI** |  |  | 0.09 |  |  |
| No | 2420 (71.6) | 1 |  | - |  |
| Yes | 37 (87.1) | 2.67 [0.86-8.31] |  | - |  |
| **Reported Stigma** |  |  | <0.001 |  | <0.001 |
| No | 346 (79.5) | 1.40 [1.01-1.93] |  | 1.59 [1.14-2.21] |  |
| Yes | 1809 (73.6) | 1 |  | 1 |  |
| Don't know | 302 (52.2) | 0.39 [0.28-0.55] |  | 0.47 [0.32-0.67] |  |
| **Discriminatory Attitudes** |  |  | 0.35 |  |  |
| No | 1760 (72.5) | 1 |  | - |  |
| Yes | 697 (70.0) | 0.89 [0.69-1.14] |  | - |  |
| **Comprehensive Knowledge about HIV** |  |  | <0.001 |  | 0.55 |
| No | 1322 (67.1) | 1 |  | 1 |  |
| Yes | 1135 (76.9) | 1.63 [1.27-2.09] |  | 1.09 [0.82-1.44] |  |
| **Exposure to TV** |  |  | 0.009 |  | 0.34 |
| Not at all | 1512 (68.4) | 1 |  | 1 |  |
| Less than once a week | 146 (72.9) | 1.24 [0.84-1.83] |  | 0.80 [0.50-1.27] |  |
| At least once a week | 799 (76.7) | 1.52 [1.16-1.98] |  | 0.77 [0.53-1.11] |  |
| **Exposure to Radio** |  |  | 0.02 |  | 0.29 |
| Not at all | 1373 (69.3) | 1 |  | 1 |  |
| Less than once a week | 304 (71.3) | 1.10 [0.76-1.59] |  | 1.08 [0.74-1.58] |  |
| At least once a week | 780 (76.1) | 1.41 [1.10-1.79] |  | 1.25 [0.95-1.66] |  |
| **Owns mobile phone** |  |  | <0.001 |  | <0.001 |
| No | 1218 (60.3) | 1 |  | 1 |  |
| Yes | 1239 (81.4) | 2.88 [2.30-3.60] |  | 1.86 [1.39-2.49] |  |
| *Use of internet* |  |  | <0.001 |  | 0.02 |
| Not at all | 2103 (70.0) | 1 |  | 1 |  |
| Less than once a week | 57 (59.6) | 0.63 [0.33-1.23] |  | 0.38 [0.19-0.77] |  |
| At least once a week | 112 (88.1) | 3.17 [1.72-5.23] |  | 1.72 [0.93-3.21] |  |
| Almost everyday | 185 (83.1) | 2.10 [1.34-3.30] |  | 1.01 [0.60-1.72] |  |

Table S3. Determinants of HIV testing uptake among sexually active young men aged 15-24, Zambia DHS 2018 (N=3114).

| **Repondent characteristics** | **N  (% coverage)** | **Crude OR (95% CI)** | **P-value** | **Adjusted OR (95% CI)** | **P-value** |
| --- | --- | --- | --- | --- | --- |
| **Age** |  |  | <0.001 |  | <0.001 |
| 15-19 | 1347 (45.5) |  |  | 1 |  |
| 20-24 | 1767 (65.8) | 2.30 [1.95-2.72] |  | 1.59 [1.29-1.96] |  |
| **Education level** |  |  | <0.001 |  | 0.01 |
| No education | 82 (52.8) | 1 |  | 1 |  |
| Primary | 1179 (46.5) | 0.78 [0.44-1.38] |  | 0.73 [0.45-1.19] |  |
| Secondary | 1758 (64.1) | 1.59 [0.92-2.77] |  | 1.05 [0.64-1.71] |  |
| Higher | 95 (67.5) | 1.86 [0.85-4.07] |  | 0.77 [0.34-1.71] |  |
| **Wealth Index** |  |  | <0.001 |  | 0.003 |
| Poorest | 619 (46.9) | 1 |  | 1.00 |  |
| Poorer | 647 (51.8) | 1.22 [0.94-1.58] |  | 1.31 [0.98-1.75] |  |
| Middle | 724 (58.9) | 1.62 [1.25-2.11] |  | 1.59 [1.16-2.20] |  |
| Richer | 561 (66.0) | 2.20 [1.49-3.25] |  | 1.45 [0.99-2.13] |  |
| Richest | 563 (61.5) | 1.81 [1.38-2.37] |  | 0.94 [0.57-1.55] |  |
| **Relationship status** |  |  | <0.001 |  | <0.001 |
| Never in union | 2564 (54.1) | 1 |  | 1 |  |
| Currently in union | 514 (74.0) | 2.42 [1.85-3.15] |  | 2.91 [2.16-3.92] |  |
| Formerly in union | 36 (65.1) | 1.59 [0.70-3.62] |  | 1.93 [0.78-4.80] |  |
| **Residence** |  |  | <0.001 |  | 0.08 |
| Urban | 1039 (65.4) | 1 |  | 1 |  |
| Rural | 2075 (52.4) | 0.58 [0.45-0.75] |  | 0.71 [0.49-1.04] |  |
| **Region** |  |  | <0.001 |  | 0.02 |
| Central | 317 (57.8) | 0.91 [0.62-1.33] |  | 1.21 [0.78-1.88] |  |
| Copperbelt | 287 (60.1) | 1 |  | 1 |  |
| Eastern | 447 (56.5) | 0.86 [0.60-1.24] |  | 1.43 [0.92-2.24] |  |
| Luapula | 341 (45.2) | 0.55 [0.37-0.82] |  | 0.89 [0.56-1.41] |  |
| Lusaka | 303 (65.2) | 1.24 [0.86-1.79] |  | 1.10 [0.72-1.68] |  |
| Muchinga | 236 (45.3) | 0.55 [0.34-0.88] |  | 0.78 [0.45-1.35] |  |
| Northern | 245 (48.9) | 0.64 [0.41-0.98] |  | 0.96 [0.57-1.62] |  |
| North western | 290 (55.0) | 0.81 [0.54-1.23] |  | 1.04 [0.65-1.65] |  |
| Southern | 348 (62.7) | 1.12 [0.61-2.04] |  | 1.35 [0.72-2.51] |  |
| Western | 300 (60.3) | 1.01 [0.70-1.45] |  | 1.65 [1.06-2.57] |  |
| **Age at first sex** |  |  | <0.001 |  | 0.50 |
| Befor 16 years | 1425 (53.2) | 1 |  | 1 |  |
| At 16 years and above | 1689 (60.7) | 1.36 [1.15-1.60] |  | 0.94 [0.78-1.13] |  |
| *Condom used* |  |  | <0.001 |  | <0.001 |
| No | 2119 (52.5) | 1 |  | 1 |  |
| Yes | 995 (67.4) | 1.87 [1.54-2.27] |  | 1.64 [1.32-2.04] |  |
| **Number of sexual partners** |  |  | <0.001 |  | 0.01 |
| None | 652 (45.7) | 1 |  | 1 |  |
| One | 1934 (61.2) | 1.87 [1.52-2.31] |  | 1.43 [1.11-1.84] |  |
| Two or more | 528 (58.4) | 1.67 [1.24-2.24] |  | 1.09 [0.79-1.51] |  |
| **History of STI** |  |  | 0.47 |  |  |
| No | 2960 (57.6) | 1 |  | - |  |
| Yes | 154 (54.1) | 0.87 [0.59-1.27] |  | - |  |
| **Circumcised** |  |  | <0.001 |  | 0.002 |
| No | 1803 (53.6) | 1 |  | 1 |  |
| Yes | 1311 (63.1) | 1.48 [1.23-1.78] |  | 1.40 [1.13-1.74] |  |
| **Reported Stigma** |  |  | <0.001 |  | 0.004 |
| No | 330 (47.6) | 0.62 [0.47-0.83] |  | 0.64 [0.47-0.86] |  |
| Yes | 2490 (59.5) | 1 |  | 1 |  |
| know/Never heard about HIV | 294 (48.9) | 0.65 [0.49-0.87] |  | 0.73 [0.54-0.99] |  |
| **Discriminatory Attitudes** |  |  | <0.001 |  | 0.11 |
| No | 2199 (60.8) | 1 |  | 1 |  |
| Yes | 915 (49.7) | 0.64 [0.52-0.78] |  | 0.84 [0.67-1.04] |  |
| **Comprehensive knowledge of HIV Know** |  |  | 0.01 |  | 0.56 |
| No | 1803 (55.2) | 1 |  | 1 |  |
| Yes | 1311 (60.5) | 1.24 [1.05-1.47] |  | 0.94 [0.77-1.15] |  |
| **Owns a mobile phone** |  |  | <0.001 |  | 0.08 |
| No | 1240 (47.2) | 1 |  | 1 |  |
| Yes | 1874 (60.2 | 1.95 [ 1.61-2.36] |  | 1.20 [0.98-1.47] |  |
| **Use of internet** |  |  | <0.001 |  | 0.003 |
| Not at all | 2365 (52.4) | 1 |  | 1 |  |
| Less than once a week | 154 (64.4) | 1.64 [1.13-2.38] |  | 1.43 [0.94-2.18] |  |
| At least once a week | 297 (71.6) | 2.29 [1.65-3.18] |  | 1.72 [1.18-2.51] |  |
| Almost everyday | 298 (73.5) | 2.52 [1.88-3.37] |  | 1.87 [1.28-2.73] |  |
| **Exposure to Television** |  |  | <0.001 |  | 0.86 |
| Not at all | 1628 (51.9) | 1 |  | 1 |  |
| Less than once a week | 384 (61.6) | 1.49 [1.06-2.10] |  | 1.08 [0.81-1.45] |  |
| At least once a week | 1102 (63.0) | 1.56 [1.33-1.88] |  | 1.01 [0.78-1.31] |  |
| **Exposure to Radio** |  |  | 0.001 |  | 0.07 |
| Not at all | 1159 (52.3) | 1 |  | 1 |  |
| Less than once a week | 476 (56.2) | 1.17 [0.93-1.46] |  | 0.98 [0.76-1.25] |  |
| At least once a week | 1479 (61.5) | 1.46 [1.22-1.73] |  | 1.24 [1.00-1.53] |  |
